# Patients' Preferences for Parkinson's Disease Pharmacotherapy: An Online Discrete Choice Experiment

**DOI:** 10.1155/padi/9526138

**Published:** 2025-07-29

**Authors:** Noriko Nishikawa, Yuki Kogo, Takayuki Ishida, Kazushi Takahashi, Atsushi Takeda

**Affiliations:** ^1^Department of Neurology, Juntendo University School of Medicine, Bunkyo, Tokyo, Japan; ^2^Medical Headquarters, Eisai Co., Ltd., Bunkyo, Tokyo, Japan; ^3^Department of Neurology, Tokyo Metropolitan Neurological Hospital, Fuchu, Tokyo, Japan; ^4^Department of Neurology, National Hospital Organization Sendai Nishitaga Hospital, Sendai, Miyagi, Japan; ^5^Department of Cognitive and Motor Aging, Tohoku University Graduate School of Medicine, Sendai, Miyagi, Japan

**Keywords:** discrete choice experiment, drug therapy, Parkinson's disease, patient preference

## Abstract

**Background:** There are many pharmacological treatment options beyond levodopa for Parkinson's disease (PD), with a variety of drug classes and formulations available. To achieve patient-centered care, clinicians must consider patients' backgrounds and preferences when selecting medications.

**Objectives:** To investigate medication preferences regarding efficacy, safety, dosage/formulation, and cost in Japanese PD patients.

**Methods:** Adults (18–90 years) from the Japan Parkinson's Disease Association receiving PD medication were eligible. An online survey was conducted, involving a discrete choice experiment, which set five medication characteristics including improvement of bothersome symptoms, risk of dyskinesia, risk of side effects other than dyskinesia, dosage/formulation, and monthly out-of-pocket cost. A questionnaire about the value of efficacy and safety of PD medications was also included.

**Results:** In the full analysis set (*N* = 207), the mean age was 65.2 years, 53.1% were female, and 62.8% had wearing-off. The most impotrant characteristics of PD medications for patients were the risk of dyskinesia, improvement of bothersome symptoms, and risk of side effects other than dyskinesia. Latent class analysis identified three groups with different preferences who have varied backgrounds, such as disease severity. The three most important symptoms patients wanted to improve were moving difficulty/slow movement (79.7%), body stiffness (43.5%), and pain (42.0%). The three most important side effects patients wanted to avoid were dyskinesia (54.6%), hallucinations/visual hallucinations (19.3%), and constipation (11.6%).

**Conclusion:** PD patients placed the highest importance on the risk of dyskinesia for PD medications and also efficacy. To achieve patient-centered care, clinicians should consider patients' backgrounds and preferences when selecting medications.

## 1. Introduction

Patient-centered care has gained attention in the field of Parkinson's disease (PD), and to achieve patient-centered care, it is essential to incorporate patients' preferences, values, and beliefs into decision-making [1, 2]. The National Institute for Health and Care Excellence (NICE) guidance for PD treatment also indicates that, before starting treatment or adding adjunctive medications, the patient's lifestyle, preferences, needs, and goals should be discussed between the patient and the physician [3]. However, there have been reports of gaps in treatment decisions and perceptions of care between PD patients and their physicians in clinical practice [4, 5], suggesting that there is room for improvement in communication. The implementation of patient-centered care is expected to improve treatment satisfaction in PD patients, which is also related to quality of life (QOL) [6, 7]. Furthermore, treatment satisfaction has been shown to influence medication adherence and treatment continuation in diseases such as cancer and mental disorders [8, 9]. Medication adherence in PD patients has been reported to be low, ranging from 10% to 67%, which is a challenge in PD pharmacotherapy [10]. Therefore, it is important to understand PD patients' preferences for improving treatment satisfaction, QOL, and medication adherence.

Levodopa represents the gold standard for PD treatment. However, patients receiving long-term treatment with levodopa frequently experience wearing-off. For wearing-off in advanced PD, adjunctive medications are added to levodopa, which may be classified into several categories based on their mechanism of action. In Japan, various types of drugs are available, including dopamine agonists (DAs), monoamine oxidase B (MAO-B) inhibitors, catechol-O-methyltransferase (COMT) inhibitors, and adenosine A2A receptor antagonists [11, 12]. Adjunctive medications improve motor symptoms and wearing-off, but they also carry the risk of side effects such as dyskinesia, drowsiness, and orthostatic hypotension [13]. In addition, there are multiple treatment options with various dosages and formulations, even within the same drug class. It is thought that patients' preferences for PD medication characteristics, including efficacy and safety, and which type of dosage/formulation, can vary for each patient. However, to the best of our knowledge, no studies have examined PD patients' preferences regarding multiple factors of pharmacotherapy in PD.

Conjoint analysis, particularly discrete choice experiments (DCEs), is used to quantify patient, caregiver, or physician preferences regarding treatment in the medical field [14]. Therefore, we investigate patients' preferences for pharmacotherapy in PD using DCE. We also evaluated what symptoms PD patients seek to improve with their PD medications and the side effects of most concern.

## 2. Materials and Methods

### 2.1. Study Design

This was a cross-sectional observational study. Members of the Japan Parkinson's Disease Association were invited to participate in the study via email from January 18 to February 13, 2024 (UMIN000053326). The questionnaire is presented in Supporting Table 1. The study was approved by the MINS Research Ethics Committee (MINS-REC-240203) and the Eisai Co., Ltd. Ethics Committee (REP-2023-0900-003-E). All patients answered the questionnaire after providing web-based consent to participate in the study.

### 2.2. Participants

Adults aged 18–90 years with diagnosed PD were eligible if they were currently receiving PD medication. If patients had trouble answering the questionnaire due to their PD symptoms or cognitive impairment, family members or caregivers were permitted to enter the answer instead of the patients. Patients who were unable to complete the web-based questionnaire or were deemed unsuitable by the investigator were excluded from the study.

### 2.3. DCE

In the DCE, two or more different objectives' profiles were simultaneously presented in the questionnaire and respondents were asked to choose the preferred one [14]. In this study, five attributes were set as PD medication characteristics: improvement of bothersome symptoms (efficacy), risk of dyskinesia (safety, dyskinesia), risk of side effects other than dyskinesia (safety, other side effects), dosage/formulation, and monthly out-of-pocket cost (cost). These five attributes were selected with the study background of optimizing current pharmacotherapy in clinical practice. Attributes considered to be determinants of drug selection were chosen based on the characteristics of anti-PD drugs that are commonly used in Japan. Levels for each attribute were set based on clinical trial data, the currently available dosage form, and prices in Japan (Table 1). Three neurologists with clinical experience, who are also authors of this study, reviewed the attributes and levels, including the expressions of the levels.

**Figure 1:**
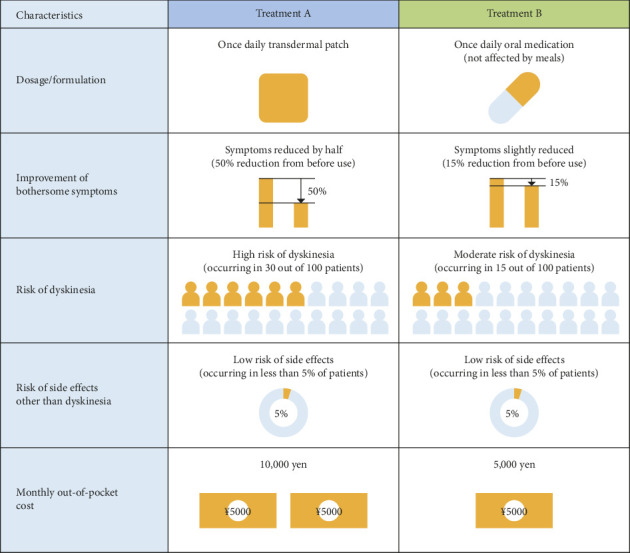
The example of the discrete choice experiment questionnaire. The questionnaire displayed two medication profiles concurrently. Patients selected their preference on the assumption that they must receive a new drug due to deteriorating PD symptoms. This questionnaire was used to evaluate patients' understanding of the survey.

An example question is presented in Figure 1. Respondents chose the preferred profile out of the two under the assumption that they must add a new drug due to deterioration of the disease. Each respondent answered 12 out of 72 questions (blocking), which were randomly ordered.

The setting and distribution of the questionnaire and sample size settings are described in the Supporting Information.

### 2.4. Value for the Efficacy and Safety of Treatments

For the symptoms that patients want to improve with medication (efficacy), respondents selected up to three symptoms out of the following seven symptoms of (1) tremor, (2) moving difficulty/slow movement, (3) body stiffness, (4) walking difficulty, (5) freezing of gait, (6) depressed mood, and (7) pain. For the side effects patients wished to avoid in medication (safety), respondents ranked three out of the following seven side effects of (1) dyskinesia, (2) daytime sleepiness, (3) dizziness, (4) hallucinations/visual hallucinations, (5) nausea, (6) edema, and (7) constipation.

### 2.5. Statistical Analysis

Descriptive statistics were used to summarize respondents' characteristics.

For the DCE, responses were coded using dummy coding. DCE data were analyzed using a conditional logit model implemented in the survival package in R because we conducted the study using blocking design. All generalized variance inflation factors were less than 3, so multicollinearity was not adjusted. Regression coefficients of each level were estimated based on the reference level, the most favorable level in each categorical attribute (Table 1). Preference weights, which quantified how much each level influences preference in the overall population, were calculated as the differences between the coefficient of each level and the mean coefficient in each attribute.

Relative attribute importance and marginal willingness to pay were calculated from the preference weights. Detailed statistical methodology has been described in the Supporting Information.

The percentage of patients selecting each symptom for improvement was calculated for the value of treatment efficacy. For safety, the percentage of patients selecting each side effect as their top 1–3 concerns was calculated. Each side effect was scored (1st place = 3 points, 2nd place = 2 points, 3rd place = 1 point), and the total score for each symptom was calculated.

All statistical analyses were performed using R software (version 4.1.1). A significance level of 5% (two-sided) and a confidence level of 95% (two-sided) were used unless otherwise specified.

### 2.6. Latent Class Analysis and Subgroup Analysis

Latent class analysis is used to classify data into subgroups with common characteristics, based on categorical qualitative data as latent variables [15]. Latent class analysis was performed using the flexmix package in R to cluster patients based on patients' preference for PD pharmacotherapy in DCE questionnaires. In this study, because participants answered a part of the DCE questionnaire, the class to which each patient belonged and regression coefficients were simultaneously estimated using the limited class mixed logit model, not considering covariates. The number of classes for latent class analysis was determined based on both Akaike Information Criterion and Bayesian Information Criterion (Supporting Table 2) according to the previous report [16]. The patient backgrounds of each class were then examined.

Subgroup analyses were also conducted for each outcome in the DCE.

## 3. Results

A total of 208 responses were collected; after excluding one duplicate response, 207 patients comprised the full analysis set. The mean age was 65.2 years (range; 39–85); 53.1% of respondents were female, and 62.8% had wearing-off (Table 2).

### 3.1. Preference Weights and Relative Attribute Importance

**Figure 2:**
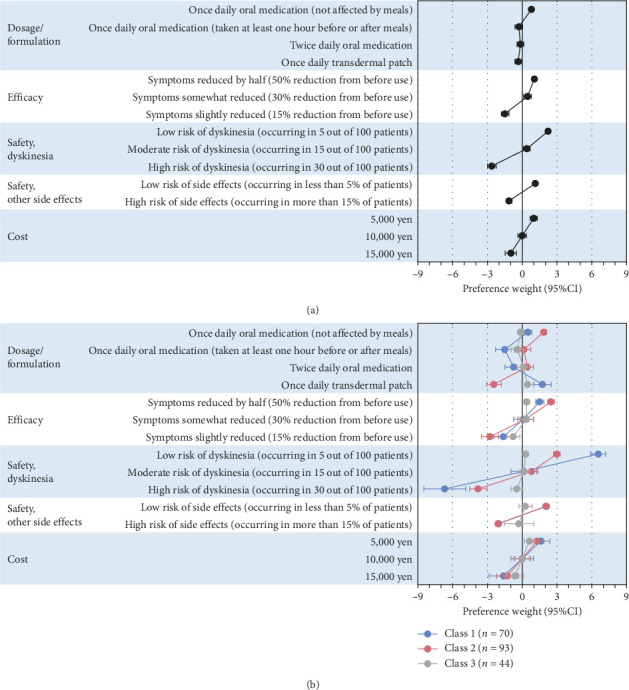
Preference weights. The upper figure shows the preference weights of the overall population (a), and the lower one shows those of the latent class analysis (b). Error bars indicate 95% confidential intervals. The plot on the right side indicates a positive impact on preferences, while the plot on the left side indicates a negative impact on preferences.

For each attribute, the regression coefficients of each level showed a significant difference from the reference level (*p* < 0.001) (Supporting Table 3). The statistical significance remained unchanged after applying the Bonferroni correction (adjusted threshold: 0.05/9 = 0.0056). Considering the 95% confidence interval, comparisons between levels also showed significant differences, with the exception of dosage/formulation. Higher efficacy levels, lower risk of side effect levels, and lower cost levels were preferred in each attribute. Dosage/formulation had a smaller impact on preferences compared to other attributes, but once-daily oral medications not affected by meals were preferred over other levels (Figure 2(a)).

**Figure 3:**
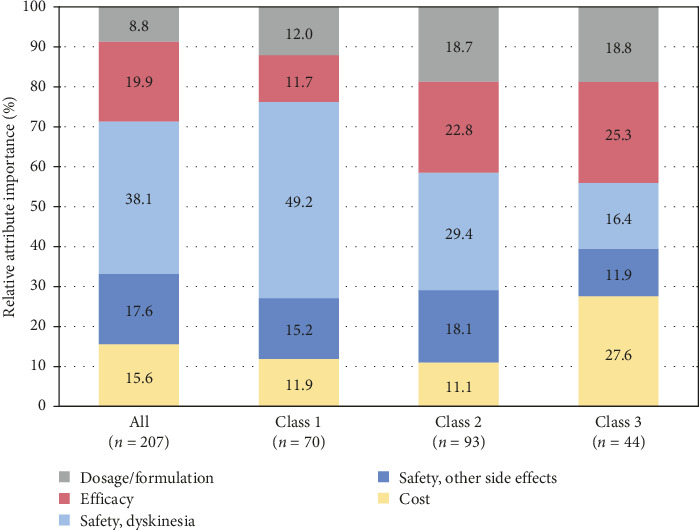
Relative attribute importance. The bar graph furthest on the left shows preference weights in the overall population, and the other three show in the latent class analysis. These values calculated from preference weights. Each component is shown as dosage/formulation, efficacy, safety (dyskinesia), safety (other side effects), and cost from top to bottom.

The most important attribute was the risk of dyskinesia (38.1%), followed by improvement of bothersome symptoms (19.9%) and the risk of side effects other than dyskinesia (17.6%) (Figure 3).

The marginal willingness to pay to switch from a medication with a high risk of dyskinesia (30% frequency) to one with a low risk (5% frequency) was approximately twice as high as that for switching from a medication with a slight symptom reduction (15% reduction) to one with a 50% reduction in symptoms (Supporting Table 4).

### 3.2. Values for Efficacy and Safety of Medications

**Figure 4:**
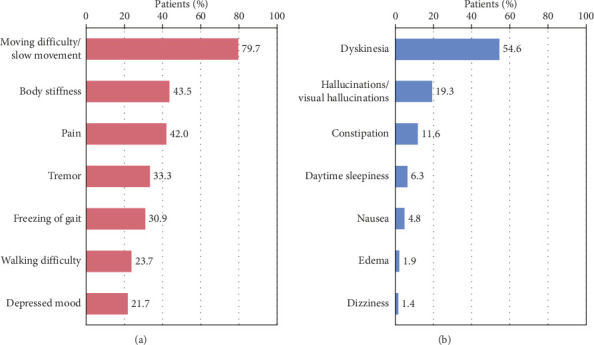
Values for efficacy and safety of medications. The horizontal axis shows the percentage of respondents who chose the symptoms. (a) The results related to efficacy. (b) The results related to safety, specifically the symptoms that patients ranked as their top concern.

The three most important symptoms that patients wanted to improve were moving difficulty/slow movement (79.7% [*n* = 165]), body stiffness (43.5% [*n* = 90]), and pain (42.0% [*n* = 87]) (Figure 4(a)). The three most important side effects patients wanted to avoid were dyskinesia (54.6% [*n* = 113]), hallucinations/visual hallucinations (19.3% [*n* = 40]), and constipation (11.6% [*n* = 24]) (Figure 4(b)). Similar results were obtained in the weighted scores for safety preferences (Supporting Table 5).

### 3.3. Latent Class Analysis

Regarding the relative attribute importance of each class, Class 1 placed a very high importance on the risk of dyskinesia compared to the overall population. Class 2 valued not only the risk of dyskinesia but also other attributes, excluding cost. Class 3 prioritized cost and improvement of bothersome symptoms over the risk of dyskinesia (Figure 3).

Regarding preference weights, Class 1 preferred transdermal patches to oral medication, whereas Class 2 preferred once-daily oral medications not affected by meals to other levels in dosage and formulation. Class 3 showed generally smaller preference weights in all attributes than the other two classes (Figure 2(b)).

For patient backgrounds, Class 1 had a higher proportion of patients with shorter disease durations, fewer types of medications other than levodopa, higher use of transdermal patches, and a higher proportion of patients who were employed or driving. Classes 2 and 3 had more patients experiencing wearing-off and dyskinesia compared to Class 1. Additionally, Class 3 had a higher proportion of patients with disease durations of ≥ 15 years. The proportion of patients treated with device-aided therapy was comparable between Classes 2 and 3 (Table 2).

The symptoms patients most wanted to improve in each class were moving difficulty/slow movement. Class 1 ranked tremor second, Class 2 ranked pain second, and Class 3 ranked walking difficulty fourth (Supporting Table 6(a)). There were no significant differences across all classes with respect to the most undesirable side effects (Supporting Table 6(b)).

### 3.4. Subgroup Analysis

The importance of each attribute was confirmed for each subgroup based on age, sex, disease duration, and presence of dyskinesia. Overall, there were no major differences in the relative attribute importance by baseline characteristics (Supporting Figure 1).

## 4. Discussion

In the pharmacotherapy of PD, understanding patient preferences is crucial for selecting the most appropriate medication from various options and achieving patient-centered care. The study aimed to evaluate patient preferences for multiple characteristics of PD medications using DCE. DCE can quantitively evaluate what respondents prioritize in multiple factors of objects. There are some studies using DCE to evaluate patients' or physicians' preference for PD treatment [17, 18]. Unlike previous studies, this study was the first to evaluate patients' preference for four factors of PD pharmacotherapy. In addition, the DCE survey did not include open-ended questions to directly query respondents' preferences; therefore, we believe that our study may reveal patients' subconscious perceptions of PD medication.

Importantly, the results of the DCE, as well as the survey to evaluate value for safety, revealed that the risk of dyskinesia had the most significant impact on patients' preference. Dyskinesia is frequent in PD patients and has an influence on their QOL [19]. However, there are few treatment options for dyskinesia within the current PD treatment options [20], which may have affected the result. Our findings suggest a high demand for treatments with a low risk of dyskinesia.

It is also worth noting that our results differed from a previous study conducted in 2008 by Hattori et al., who reported that constipation was the most concerning side effect for Japanese patients, with dyskinesia ranking eighth [21]. Firstly, the difference in findings may reflect the increased awareness of dyskinesia in our study. People can more easily access information via the internet now compared with over 10 years ago. Additionally, this survey was conducted online, and respondents were likely to be more information-literate and proactive in seeking information about their condition, resulting in greater awareness of dyskinesia. Furthermore, treatment regimens have changed over the past few decades. In the 2000s, initiation of DA monotherapy was often recommended as first-line PD treatment. However, the results of several clinical trials have led to the recognition of levodopa as a first-line drug. This may have increased the opportunity for physicians to explain levodopa-induced dyskinesia, resulting in increased patient awareness of dyskinesia. Secondly, this gap may be due to the difference in treatment options. There are more treatment options now available affording better control of symptoms than previously. This has led to an increased emphasis from patients on improvement of QOL, and patients are now more concerned about dyskinesia compared with a few decades ago. Finally, the difference may be caused by respondents' characteristics. The patients of this survey were younger and therefore more likely to be independent and thus employed or driving, which may also contribute to their greater concern about dyskinesia impacting their daily living activities.

This study showed that patients placed the highest importance on the risk of dyskinesia, but they prioritized efficacy of treatment as secondary. Dyskinesia is inextricably linked to the efficacy of PD pharmacotherapy. When a patient and their physician make a decision regarding treatment, it is important to explain both the efficacy and safety of drugs and to decide treatment with mutual consent.

The most important symptom patients wanted to improve was moving difficulty/slow movement not only in the overall population but also in each latent class. This result highlights the importance of addressing bradykinesia to enhance patient satisfaction with PD treatment. Patients also wanted to improve body stiffness and pain. These three most important symptoms are very prevalent and are difficult to control with medication [22], which may explain their high ranking. These findings show that while the primary expectation is to improve motor symptoms, there is also a significant demand for improving nonmotor symptoms, pointing to the need for comprehensive symptom management.

Latent class analysis was exploratorily performed to analyze the diversity of patient preferences. The analysis suggested the potential existence of three groups with different preferences and varied patient backgrounds. Class 1 tended to place a very high importance on the risk of dyskinesia despite having a lower proportion of patients experiencing dyskinesia. Previous reports have suggested that patients who have not experienced dyskinesia tend to be more concerned about its risk, which was also observed in this study [23]. Class 2 showed a tendency to value not only the risk of dyskinesia but also efficacy, other side effects, and dosage/formulation. This class is likely to consist of patients whose disease has progressed to some extent but whose symptoms are relatively well controlled with treatment considering the severity and the usage of device-aided therapy. Class 3 leaned toward prioritizing cost and efficacy over the risk of dyskinesia. This class likely includes patients with more advanced disease and a higher prevalence of wearing-off and dyskinesia. It is inferred that the disease progresses from Class 1 to Class 3, and the relative importance of dyskinesia risk decreases while the relative importance of efficacy increases with progression. This finding suggests that advanced PD patients fear severe off-states, so they prioritize avoiding off-states and improving symptoms over side effect risks, reflecting their greater experience with wearing-off and dyskinesia. In latent class analysis, the number of patients ranged from 44 to 93 in each class, and statistical power might be insufficient. No statistical test was performed for comparison between the classes; therefore, larger studies will be needed to explore patient's preference in greater depth.

The analysis of preference weights revealed that a preference for once-daily oral medication that is not affected by meals was highly valued within the dosage/formulation attribute. This preference for medications that require fewer doses aligns with the findings of Schapira et al. [24] and Serbin et al. [17]. Interestingly, formulations other than once-daily oral medications that were not affected by meals did not have a strong impact on patient preferences. Most patients with PD in this study were taking levodopa (95.2%), which is typically taken at least three times per day. This suggested that the number of doses per day may not significantly influence preferences in PD patients. In the latent class analysis, Class 2 showed lower preference weights for transdermal patches, and there were fewer patients using them compared to other classes. This suggests that the actual use and perceived effectiveness and convenience of transdermal patches influenced their preferences.

This study must also be considered in light of its limitations. Firstly, this study was conducted via a web-based survey. Respondents were required to use digital devices such as a computer or smartphone to participate in the web survey, which means that respondents were relatively information literate. There were relatively few elderly participants in this study, but the age distribution of PD patients in Japan is highest in the 80–84 age group [25]. In general, volunteer participants are considered to be highly interested in the topics of the survey. This resulted in recruiting patients with biased backgrounds, making generalization difficult. Secondly, the diverse backgrounds of the subjects, including their medication use and side effect experiences and regional factors (urban vs. suburban), could have influenced the results. In addition, this survey enrolled only Japanese patients. Patient preferences might differ among countries because they are affected by cultural factors such as personality, environment, and lifestyle. Furthermore, out-of-pocket costs vary based on Japan's healthcare and subsidy systems. These settings make it difficult to generalize the results. Thirdly, the DCE used hypothetical treatment options for quantitative analysis, which may not accurately reflect clinical practice. In addition, only five attributes were set in this study, considering the patient burden. Fourthly, although there might be some unmeasured confounders such as the past experiences or doctors' advice, we could not put them into the analysis model. Finally, the survey did not include open-ended responses, which may introduce selection bias.

## 5. Conclusions

From this study, it was evident that Japanese PD patients place the highest importance on the risk of dyskinesia when selecting PD treatments, followed by efficacy and the risk of other side effects. This study found that patients with early PD who have not experienced motor complications are most concerned about dyskinesia, suggesting that patient education is important for an appropriate understanding of treatment.

## Figures and Tables

**Table 1:** List of attributes and levels for the discrete choice experiment.

Attribute	Levels
Dosage/formulation	- *Once daily oral medication (not affected by meals)*
	- Once daily oral medication (taken at least 1 hour before or after meals)
	- Twice daily oral medication
	- Once daily transdermal patch

Improvement of bothersome symptoms	- *Symptoms reduced by half (50% reduction from before use)*
	- Symptoms somewhat reduced (30% reduction from before use)
	- Symptoms slightly reduced (15% reduction from before use)

Risk of dyskinesia	- *Low risk of dyskinesia (occurring in 5 out of 100 patients)*
	- Moderate risk of dyskinesia (occurring in 15 out of 100 patients)
	- High risk of dyskinesia (occurring in 30 out of 100 patients)

Risk of side effects other than dyskinesia	- *Low risk of side effects (occurring in less than 5% of patients)*
	- High risk of side effects (occurring in more than 15% of patients)

Monthly out-of-pocket cost	- 5000 yen
	- 10,000 yen
	- 15,000 yen

*Note:* Italic levels are reference levels.

**Table 2:** Demographics and baseline characteristics of respondents.

	Overall (*n* = 207)	Class 1 (*n* = 70)	Class 2 (*n* = 93)	Class 3 (*n* = 44)
Sex (female), *n* (%)	110 (53.1)	40 (57.1)	46 (49.5)	24 (54.5)
Age, years, mean (SD), (min - max)	65.2 (8.2), (39–85)	64.8 (7.7), (47–85)	66.5 (8.5), (39–82)	63.0 (8.2), (41–74)
Duration of PD, *n* (%)				
< 5 years	50 (24.2)	21 (30.0)	20 (21.5)	9 (20.5)
5 to < 10 years	59 (28.5)	22 (31.4)	23 (24.7)	14 (31.8)
10 to < 15 years	52 (25.1)	18 (25.7)	27 (29.0)	7 (15.9)
≥ 15 years	46 (22.2)	9 (12.9)	23 (24.7)	14 (31.8)
Modified Hoehn and Yahr stage (on state)^1^, mean	1.98	1.81	2.07	2.10
1/2	131 (63.2)	53 (75.7)	51 (54.8)	27 (61.3)
3/4/5	64 (30.9)	15 (21.4)	34 (36.6)	15 (34.1)
Unknown	12 (5.8)	2 (2.9)	8 (8.6)	2 (4.5)
Modified Hoehn and Yahr stage (off state)^1^, mean	2.98	2.69	3.05	3.33
1/2	48 (23.2)	22 (31.4)	20 (21.5)	6 (13.6)
3/4/5	141 (68.1)	46 (65.7)	62 (66.7)	33 (75.0)
Unknown	18 (8.7)	2 (2.9)	11 (11.8)	5 (11.4)
Recipients of subsidies for medical treatment of intractable diseases^2^, *n* (%)	169 (81.6)	58 (82.9)	74 (79.6)	37 (84.1)
Motor fluctuation, *n* (%)				
With wearing-off	130 (62.8)	40 (57.1)	61 (65.6)	29 (65.9)
With dyskinesia	84 (40.6)	21 (30.0)	41 (44.1)	22 (50.0)
Treatment for PD, *n* (%)				
Levodopa	197 (95.2)	64 (91.4)	90 (96.8)	43 (97.7)
Oral medication (other than levodopa)^3^	146 (70.5)	46 (65.7)	65 (69.9)	35 (79.5)
1	28 (13.5)	12 (17.1)	7 (7.5)	9 (20.5)
2	44 (21.3)	17 (24.3)	20 (21.5)	7 (15.9)
≥ 3	60 (29.0)	17 (24.3)	26 (28.0)	17 (38.6)
Unknown	14 (6.8)	0 (0.0)	12 (12.9)	2 (4.5)
Transdermal patch	84 (40.6)	36 (51.4)	28 (30.1)	20 (45.5)
Levodopa/carbidopa intestinal gel	6 (2.9)	3 (4.3)	2 (2.2)	1 (2.3)
CSCI of levodopa/carbidopa prodrugs	4 (1.9)	2 (2.9)	2 (2.2)	0 (0.0)
Brain surgery (DBS)	27 (13.0)	7 (10.0)	13 (14.0)	7 (15.9)
Number of daily oral doses, *n* (%)				
1–3/day	78 (37.7)	34 (48.6)	28 (30.1)	16 (36.4)
4–5/day	67 (32.4)	21 (30.0)	35 (37.6)	11 (25.0)
≥ 6/day	62 (30.0)	15 (21.4)	30 (32.3)	17 (38.6)
Working, *n* (%)	47 (22.7)	20 (28.6)	19 (20.4)	8 (18.2)
Driving a car, *n* (%)	88 (42.5)	36 (51.4)	36 (38.7)	16 (36.4)
Living situation, *n* (%)				
Lives alone	13 (6.3)	2 (2.9)	8 (8.6)	3 (6.8)
Lives with others^4^	192 (92.8)	68 (97.1)	84 (90.3)	40 (90.9)
Other situations	2 (1.0)	0 (0.0)	1 (1.1)	1 (2.3)
Highest level of education, *n* (%)				
Junior high or high school diploma	62 (30.0)	22 (31.4)	25 (26.9)	15 (34.1)
Associate degree	65 (31.4)	21 (30.0)	30 (32.3)	14 (31.8)
Bachelor or Master or Doctor's degree	80 (38.6)	27 (38.6)	38 (40.9)	15 (34.1)

Abbreviations: CSCI, continuous subcutaneous infusion; DBS, deep brain stimulation; PD, Parkinson's disease; SD, standard deviation.

^1^Reported by patients.

^2^The Japanese medical system. Patients who have severe PD or pay high medical costs can receive medical service subsidies.

^3^Denominator is the number of patients in the overall population or each class.

^4^Including but not limited to partners, roommates, children, and parents.

## Data Availability

The data that support the findings of this study are available from the corresponding author upon reasonable request.

## Supporting Information

Supporting Information 1 Supporting Information. The detail of discrete choice experiment methods and statistical analysis.

Supporting Information 2 Supporting Table 1: Questionnaire in English.

Supporting Information 3 Supporting Table 2: The relative performance of Akaike information criterion (AIC) and Bayesian information criterion (BIC) for model selection (Latent Class Analysis).

Supporting Information 4 Supporting Table 3: Estimated regression coefficient of conjoint analysis.

Supporting Information 5 Supporting Table 4: Willingness to pay for each attribute.

Supporting Information 6 Supporting Table 5: Ranking of treatment safety preferences.

Supporting Information 7 Supporting Table 6: Ranking of treatment (a) efficacy and (b) safety preferences in the latent class.

Supporting Information 8 Supporting Figure 1: Relative attribute importance according to various demographic and clinical characteristics.
