# Supplementary material for: Patients' Preferences for Parkinson's Disease Pharmacotherapy: An Online Discrete Choice Experiment
Source: Parkinsons Dis. 2025 Jul 29;2025:9526138. doi: 10.1155/padi/9526138 (PMC12324919; doi:10.1155/padi/9526138)
Supplement: Supporting Information 1 — Supporting Information. The detail of discrete choice experiment methods and statistical analysis. [file 9526138.f1.docx]

**Supplemental Methods**

Settings and distribution of DCE questionnaire

From the set attributes and levels, 216 drug profiles were created. 216 profiles orthogonalized using the L72 array, and a total of 72 combinations of medication profiles were established. To reduce patients’ burden, these sets of profiles were divided into 6 blocks. Respondents were assigned to one block which the fewest respondents included. When the number of answerers was the same in some blocks, each respondent was randomly assigned one of six blocks.

Sample size settings

A target sample size of at least 167 patients was estimated based on a practical guide for DCE^1^ ,which utilized the following formula:

- - N > (500 × the number of levels in the largest attribute) / (the number of questions per respondent × the number of choices per question).
  - In this study, the number of levels in the largest attribute is 4, the number of questions per respondent is 12, and the number of choices per question is 2, resulting in a calculation of N >83.3.

Considering the stratified subgroup analysis into two groups, it was calculated that a total sample size of at least 167 would be required.

Statistical analysis of DCE

1. Preference weights

The model for estimating the utility V is as follows, where βij is the coefficient of the level (j) of the attribute (i).

$$V=\beta_{1j}{Dosage}_{j}+\beta_{2j}{Symp}_{j}+\beta_{3j}{Dysk}_{j}+\beta_{4j}{Side}_{j}+\beta_{5j}Price+\varepsilon$$

The preference weight is calculated as the deviation of the regression coefficient βij for each attribute from the average value. Uij is calculated as follows, where the preference weight of the level (j) of the attribute (i) and m be the number of levels of the attribute (i).

$$U_{ij}=\beta_{ij}-\frac{\sum_{j=1}^{m} \beta_{ij}}{m}$$

2. RAI

Imi, the importance of the attribute (i), is calculated using the following formula, where Uij is the preference weight value of the level (j) of the attribute (i).

$${Im}_{i}(\%)=\frac{Max\left( U_{ij} \right)-Min\left( U_{ij} \right)}{\sum_{i=1}^{5} (Max\left( U_{ij} \right)-Min\left( U_{ij} \right))}$$

3. MWTP

The marginal willingness to pay (MWTP) is defined as the increase in cost when the level of a certain attribute is changed while keeping the determined utility V constant. The MWTP when changing the attribute (i) from the level (n) to the level (n+1) is calculated as follows. The MWTP is calculated except for cost.

$$MWTP=-\frac{\beta_{in+1}-\beta_{in}}{\beta_{5}}$$

Reference

1. de Bekker-Grob, E. W., Donkers, B., Jonker, M. F. et al. Sample size requirements for Discrete-Choice Experiments in healthcare: a practical guide. *Patient* 2015; 8: 373-384.
