# Supplementary material for: Patients' Preferences for Parkinson's Disease Pharmacotherapy: An Online Discrete Choice Experiment
Source: Parkinsons Dis. 2025 Jul 29;2025:9526138. doi: 10.1155/padi/9526138 (PMC12324919; doi:10.1155/padi/9526138)
Supplement: Supporting Information 2 — Supporting Table 1: Questionnaire in English. [file 9526138.f2.pdf]

About This Survey

This survey is subjected the patients who are diagnosed with Parkinson's disease and receive medications for its treatment at present.
Family members or caregivers may also enter the answer instead of the patients.

Q1 Please tell us your age in years.

( ) years old

Q2 We will ask about your treatment for Parkinson's disease.

1-1) Please provide information about the medication you currently receive.
For the following medications, please select whether you 'currently receive' or 'don't currently receive' each one.

		Currently receive	Don't currently receive
1	Levodopa oral medication	☐	☐
2	Oral medication other than levodopa (such as dopamine agonists [excluding herbal medicines and supplements])	☐	☐
3	Dopamine agonist transdermal patches	☐	☐

1-2) If you answered 'receive' for oral medications other than levodopa (such as dopamine agonists), how many types of medication do you currently receive?
Please select an appricable answer from the following.
Oral medications other than levodopa include dopamine agonists, MAO-B inhibitors, COMT inhibitors, etc.
Please select the applicable one, excluding traditional Chinese medicine and supplements.

1 1 type
2 2 types
3 3 or more types
4 I don't know

2) Have you ever received treatment for Parkinson's disease through surgery or medical devices, either currently or in the past?
Please select one or more appricable answers from the following.

1 Use of levodopa intestinal solution (administered directly to the small intestine through a tube in the abdomen)
2 Use of levodopa subcutaneous injection formulation (administered under the skin of the abdomen)
3 Deep brain stimulation therapy (DBS, a method of implanting electrodes in the deep parts of the brain for stimulation) and neurosurgery
4 I have never experienced any of the above treatments
5 I don't know

Q3 How many years have passed since you were diagnosed with Parkinson's disease? Please select an appricable answer from the following.

1 Less than 5 years
2 More than 5 years but less than 10 years
3 More than 10 years but less than 15 years
4 More than 15 years
5 I don't know

Q4 Please select the condition that most closely matches your current symptoms of Parkinson's disease from the following 1 to 5. If you are not sure, please select 6.
Please select an appricable condition each for when the Parkinson's disease medication is effective and when it is not.

	When the medication is effective	When the medication is not effective
① Symptoms are only in the limbs on one side, with no issues in walking or balance (Hoehn and Yahr stage 1)	☐	☐
② Symptoms are in the limbs on both sides, but there are no issues in walking or balance (Hoehn and Yahr stage 2)	☐	☐
③ Symptoms are in the limbs on both sides, with walking disabilities such as shuffling or freezing gait, and there are difficulties in maintaining balance (Hoehn and Yahr stage 3)	☐	☐
④ Symptoms are in the limbs on both sides, and it is difficult to stand up or walk in daily life without a caregiver (Hoehn and Yahr stage 4)	☐	☐
⑤ Unable to walk alone, a wheelchair is needed for mobility, and a caregiver is necessary (Hoehn and Yahr stage 5)	☐	☐
⑥ Unable to determine my own condition	☐	☐

Q5 Do you currently have a recipient certificate for the Intractable Diseases Medical Service Subsidy (specific medical expenses)? Please select one or more appricable answers from the following.

1 I have it
2 I don't have it
3 I don't know

Q6 How often do you take your medication in a day? Please select one or more appricable answers from the following.
Please answer the number of times including medications other than for Parkinson's disease, excluding transdermal patches and supplements.

1 Up to 3 times in a day (e.g. morning, noon, evening)
2 4 to 5 times in a day
3 More than 6 times in a day

Q7 Please select one option that most closely matches your relationship with your doctor treating your Parkinson's disease.

1 I can consult my doctor about anything and am satisfied with the treatment plan
2 I can somewhat consult my doctor and am somewhat satisfied with the treatment plan
3 I can't consult my doctor much and am not satisfied with some parts of the treatment plan
4 I can't consult my doctor at all and am not satisfied with the treatment plan

Q8 Please tell us your gender.

1 Male
2 Female
3 I don't want to answer

Q9 Please select one option that most closely matches your living environment.

1 Living alone
2 Living with family or partner
3 Living in a nursing home
4 Not applicable

Q10 Please tell us about your current daily life. For the following two items, please select either 'Yes' or 'No' for each.

		Yes	No
1	I work	☐	☐
2	I drive myself	☐	☐

Q11 Please select your educational background from the following options.

1 Graduated from a university or graduate school
2 Graduated from a junior college or vocational school
3 Graduated from high school or junior high school
4 Other than the above

We would like to ask about your values regarding the symptoms and treatment of Parkinson's disease.

Q1SQ Please tell us about the bothersome symptoms that you want to improve with medication. Of the following seven symptoms, please select up to three that you particularly want to improve.

	Symptoms	Symptoms to improve
1	Trembling of limbs (tremor)	☐
2	Difficulty moving, slow movement	☐
3	Stiffness in some part of the body	☐
4	Difficulty walking due to reduced stride	☐
5	Difficulty taking the first step when starting to walk (freezing of gait)	☐
6	Depressed mood	☐
7	Body pain (such as lower back pain)	☐

Q2SQ_1 The following questions are about the symptoms of Parkinson's disease and the side effects of the medication.

This is an explanation of the symptoms of Parkinson's disease and side effects.

Symptoms	Explanation
Wearing-off	A condition which Parkinson's disease symptoms fluctuate in the daytime. The improvement effect of medication on symptom does not last long, leading to apperaence of Parkinson's disease symptoms, such as difficulty moving and feelings of depression, before the next dose. You have bad times, low times, and times when the medication is not effective throughout the day.
Dyskinesia	Symptoms that body moves involuntaly without control caused by Parkinson's disease medication, such as the hands, feet, and shoulders swaying, and the mouth moving mumbling.

Regarding the following symptoms, please select whether you currently have them, do not have them, or do not know.

	Symptoms	Current symptoms		
		Present	Absent	Unknown
1	Wearing-off	☐	☐	☐
2	Dyskinesia	☐	☐	☐

Q2SQ_2 This is an explanation of the main side effects that may occur when taking the medication.

Symptoms	Explanation
Daytime sleepiness	Falling asleep while relaxing during the daytime (while reading, watching TV). If the symptoms are severe, one may fall asleep at times when they should not (such as during meals or while talking to someone).
Dizziness upon standing (orthostatic hypotension)	When standing up, there may be dizziness and lightheadedness, necessitating holding onto something. In severe cases, one may faint and collapse.
Hallucinations/visual hallucinations	Illusions, or seeing objects and creatures that don't actually have a form. For example, seeing pictures or wall patterns move. Seeing insects that don't exist, seeing people who aren't there.
Edema	Swelling of the dorsum of the foot and ankles due to poor blood flow.
Nausea	Feeling nauseous and uncomfortable.
Constipation	Being unable to fully evacuate the bowels.

For the following side effects, please select whether you have experienced them or not.

	Side effects	Experience of side effects	
		Yes	No
1	Daytime sleepiness	☐	☐
2	Dizziness upon standing (orthostatic hypotension)	☐	☐
3	Hallucinations/visual hallucinations	☐	☐
4	Edema	☐	☐
5	Nausea	☐	☐
6	Constipation	☐	☐

Q3SQ Now, we will ask about your values regarding the side effects of new Parkinson's disease medications.
Medication for Parkinson's disease can improve symptoms, but it can also have side effects.
Please select three medication side effects you wish to avoid and rank them in order of importance.

	Side effects	Ranking of side effects to avoid		
		1st	2nd	3rd
1	Dyskinesia	☐	☐	☐
2	Daytime sleepiness	☐	☐	☐
3	Dizziness upon standing (orthostatic hypotension)	☐	☐	☐
4	Hallucinations/visual hallucinations	☐	☐	☐
5	Edema	☐	☐	☐
6	Nausea	☐	☐	☐
7	Constipation	☐	☐	☐

Q4SQ From here, we will ask about your preferences regarding medication for Parkinson's disease.
Two types of medication with different profiles will be displayed.
If you need to add new medication due to the progression of Parkinson's disease symptoms, please select the one you would prefer to receive.

The medication displayed next has the following five characteristics, so please review the description before proceeding to the questions.
You can review the explanation about this medication later.

Attribute	Description of characteristics	Levels
Dosage/formulation	Indicates that the number of times medication is taken per day, the timing of medication, and the dosage form.	・Once daily oral medication (not affected by meals)<br>・Once daily oral medication (take more than 1 hour before or after meals)<br>・Twice daily oral medication

		・Once daily transdermal patch
Improvement of bothersome symptoms	Indicates how much the Parkinson's disease symptoms that you are currently having trouble with will improve by taking the medication. Please consider it as the expected average effect, although individual differences exist.	・Symptoms reduced by half (50% reduction from before use)<br>・Symptoms somewhat reduce (30% reduction from before use)<br>・Symptoms slightly reduced (15% reduction from before use)
Risk of dyskinesia	Indicates the likelihood of dyskinesia occurring by taking the medication.	・Low risk of dyskinesia (occurring in 5 out of 100 patients)<br>・Moderate risk of dyskinesia (occurring in 15 out of 100 patients)<br>・High risk of dyskinesia (occurring in 30 out of 100 patients)
Risk of side effects other than dyskinesia	Indicates the likelihood of side effects (such as drowsiness, hallucinations, nausea) occurring by taking the medication.	・Low risk of side effects (occurring in less than 5% of patients)<br>・High risk of side effects (occurring in more than 15% of patients)
Monthly out-of-pocket cost	Indicates an estimated cost you will pay if prescribed the medication for one month. Consider it as the cost needed in addition to your current payment.	・5,000 yen<br>・10,000 yen<br>・15,000 yen

Q4SQ_1 Please answer all of the following twelve multiple-choice questions, similar to the example shown next.

First, please answer the example question.

Characteristics	Treatment A	Treatment B
**Dosage/formulation**	**Once** daily **oral medication** (**not affected** by meals)	**Twice** daily **oral medication**
**Improvement of bothersome symptoms**	Symptoms reduced **by half** (**50%** reduction from before use) 50%	Symptoms **slightly** reduced (**15%** reduction from before use) 15%
**Risk of dyskinesia**	**Lower risk** of dyskinesia (occurring in **5** out of 100 patients)	**Higher risk** of dyskinesia (occurring in **30** out of 100 patients)
**Risk of side effects other than dyskinesia**	**Lower risk** of side effects (occurring in **less than 5%** of patients) 5%	**Higher risk** of side effects (occurring in **more than 15%** of patients) 15%
**Monthly out-of-pocket cost**	**5,000 yen** ¥5000	**15,000 yen** ¥5000 ¥5000 ¥5000
