# Supplementary material for: Patients' Preferences for Parkinson's Disease Pharmacotherapy: An Online Discrete Choice Experiment
Source: Parkinsons Dis. 2025 Jul 29;2025:9526138. doi: 10.1155/padi/9526138 (PMC12324919; doi:10.1155/padi/9526138)
Supplement: Supporting Information 3 — Supporting Table 2: The relative performance of Akaike information criterion (AIC) and Bayesian information criterion (BIC) for model selection (Latent Class Analysis). [file 9526138.f3.docx]

**Table S2.** The relative performance of Akaike information criterion (AIC) and Baysian information criterion (BIC) for model selection (Latent Class Analysis)

|  | AIC | BIC |
| --- | --- | --- |
| Class 1 | 2420.269 | 2478.866 |
| Class 2 | 2334.311 | 2458.016 |
| Class 3 | 2272.900 | 2461.712 |
| Class 4 | 2250.746 | 2504.666 |
