# Supplementary material for: Patients' Preferences for Parkinson's Disease Pharmacotherapy: An Online Discrete Choice Experiment
Source: Parkinsons Dis. 2025 Jul 29;2025:9526138. doi: 10.1155/padi/9526138 (PMC12324919; doi:10.1155/padi/9526138)
Supplement: Supporting Information 4 — Supporting Table 3: Estimated regression coefficient of conjoint analysis. [file 9526138.f4.docx]

**Table S3.** Estimated regression coefficient of conjoint analysis

| **Attribute** | **Level** | **Estimated regression coefficient** | **Exponentially transformed regression coefficients**  **(95% confidential intervals)** | **Z score** | **p-value** |
| --- | --- | --- | --- | --- | --- |
| Dosage/formulation | Once daily oral medication (not affected by meals) | reference | - | - | - |
|  | Once daily oral medication (taken at least one hour before or after meals) | -1.0777 | 0.3404  (0.2510, 0.4614) | -6.9351 | <0.001* |
|  | Twice daily oral medication | -0.9498 | 0.3868  (0.2954, 0.5065) | -6.9064 | <0.001* |
|  | Once daily transdermal patch | -1.1177 | 0.3270  (0.2517, 0.4249) | -8.3661 | <0.001* |
| Improvement in bothersome symptoms | Symptoms reduced by half | reference | - | - | - |
|  | Symptoms somewhat reduced | -0.5872 | 0.5559  (0.4068, 0.7595) | -3.6872 | <0.001* |
|  | Symptoms slightly reduced | -2.5224 | 0.0803  (0.0596, 0.1082) | -16.5666 | <0.001* |
| Risk of dyskinesia | Low risk of dyskinesia | reference | - | - | - |
|  | Moderate risk of dyskinesia | -1.8341 | 0.1598  (0.1240, 0.2058) | -14.1895 | <0.001* |
|  | High risk of dyskinesia | -4.8301 | 0.0080  (0.0057, 0.0112) | -27.6902 | <0.001* |
| Risk of side effects other than dyskinesia | Low risk of side effects | reference | - | - | - |
|  | High risk of side effects | -2.2341 | 0.1071  (0.0862, 0.1330) | -20.1728 | <0.001* |
| Monthly out-of-pocket cost | - | -0.0002 | 0.9998  (0.9998, 0.9998) | -14.3178 | <0.001* |
