# Supplementary material for: Patients' Preferences for Parkinson's Disease Pharmacotherapy: An Online Discrete Choice Experiment
Source: Parkinsons Dis. 2025 Jul 29;2025:9526138. doi: 10.1155/padi/9526138 (PMC12324919; doi:10.1155/padi/9526138)
Supplement: Supporting Information 5 — Supporting Table 4: Willingness to pay for each attribute. [file 9526138.f5.docx]

**Table S4.** Willingness to pay for each attribute

| **Attribute** | **To** | **From** | **Willing to pay** |
| --- | --- | --- | --- |
| **Dosage/formulation** | Once daily oral medication (not affected by meals) | Once daily oral medication (taken at least one hour before or after meals) | 5,454 yen |
|  |  | Twice daily oral medication | 4,807 yen |
|  |  | Once daily transdermal patch | 5,657 yen |
| **Improvement of bothersome symptoms** | Symptoms reduced by half (50% reduction from before use) | Symptoms somewhat reduced (30% reduction from before use) | 2,972 yen |
|  |  | Symptoms slightly reduced (15% reduction from before use) | 12,767 yen |
| **Risk of dyskinesia** | Low risk of dyskinesia (occurring in 5 out of 100 patients) | Moderate risk of dyskinesia (occurring in 15 out of 100 patients) | 9,283 yen |
|  |  | High risk of dyskinesia (occurring in 30 out of 100 patients) | 24,446 yen |
| **Risk of other side effects** | Low risk of side effects (occurring in less than 5% of patients) | High risk of side effects (occurring in more than 15% of patients) | 11,307 yen |
