# Supplementary material for: Patients' Preferences for Parkinson's Disease Pharmacotherapy: An Online Discrete Choice Experiment
Source: Parkinsons Dis. 2025 Jul 29;2025:9526138. doi: 10.1155/padi/9526138 (PMC12324919; doi:10.1155/padi/9526138)
Supplement: Supporting Information 6 — Supporting Table 5: Ranking of treatment safety preferences. [file 9526138.f6.docx]

**Table S5.** Ranking of treatment safety preferences

| **Symptoms** | **Rank** | | | **Score** |
| --- | --- | --- | --- | --- |
|  | **1**  **N (%)** | **2**  **N (%)** | **3**  **N (%)** |  |
| Dyskinesia | 113 (54.6) | 39 (18.8) | 20 (9.7) | 437 |
| Hallucinations/Visual hallucinations | 40 (19.3) | 50 (24.2) | 26 (12.6) | 246 |
| Constipation | 24 (11.6) | 36 (17.4) | 49 (23.7) | 193 |
| Daytime sleepiness | 13 (6.3) | 43 (20.8) | 41 (19.8) | 166 |
| Nausea | 10 (4.8) | 14 (6.8) | 28 (13.5) | 86 |
| Edema | 4 (1.9) | 2 (1.0) | 15 (7.2) | 83 |
| Dizziness | 3 (1.4) | 23 (11.1) | 28 (13.5) | 31 |
