# Supplementary material for: Patients' Preferences for Parkinson's Disease Pharmacotherapy: An Online Discrete Choice Experiment
Source: Parkinsons Dis. 2025 Jul 29;2025:9526138. doi: 10.1155/padi/9526138 (PMC12324919; doi:10.1155/padi/9526138)
Supplement: Supporting Information 7 — Supporting Table 6: Ranking of treatment (a) efficacy and (b) safety preferences in the latent class. [file 9526138.f7.docx]

**Table S6.** Ranking of treatment a) efficacy and b) safety preferences in the latent class

A)

| **Rank in the  overall population** | **Symptoms** | Class 1 (n=70) | | | Class 2 (n=93) | | | Class 3 (n=44) | | |
| --- | --- | --- | --- | --- | --- | --- | --- | --- | --- | --- |
|  |  | n | % | Rank | n | % | Rank | n | % | Rank |
| 1 | Moving difficulty/ slow movement | 58 | 82.9 | 1 | 70 | 75.3 | 1 | 37 | 84.1 | 1 |
| 2 | Body stiffness | 30 | 42.9 | 2 | 41 | 44.1 | 3 | 19 | 43.2 | 2 |
| 3 | Pain | 24 | 34.3 | 4 | 46 | 49.5 | **2** | 17 | 38.6 | 3 |
| 4 | Tremor | 30 | 42.9 | **2** | 28 | 30.1 | 5 | 11 | 25.0 | 6 |
| 5 | Freezing of gait | 21 | 30.0 | 5 | 30 | 32.3 | 4 | 13 | 29.5 | 5 |
| 6 | Walking difficulty | 15 | 21.4 | 6 | 19 | 20.4 | 7 | 15 | 34.1 | **4** |
| 7 | Depressed mood | 15 | 21.4 | 6 | 22 | 23.7 | 6 | 8 | 18.2 | 7 |

B)

| **Rank in the overall population** | **Symptoms** | Class 1 (n=70) | | | Class 2 (n=93) | | | Class 3 (n=44) | | |
| --- | --- | --- | --- | --- | --- | --- | --- | --- | --- | --- |
|  |  | n | % | Rank | n | % | Rank | n | % | Rank |
| 1 | Dyskinesia | 45 | 64.3 | 1 | 44 | 47.3 | 1 | 24 | 54.5 | 1 |
| 2 | Hallucinations/ visual hallucinations | 10 | 14.3 | 2 | 23 | 24.7 | 2 | 7 | 15.9 | 2 |
| 3 | Constipation | 6 | 8.6 | 3 | 12 | 12.9 | 3 | 6 | 13.6 | 3 |
| 4 | Daytime sleepiness | 3 | 4.3 | 5 | 8 | 8.6 | 4 | 2 | 4.5 | 5 |
| 5 | Nausea | 4 | 5.7 | 4 | 3 | 3.2 | 5 | 3 | 6.8 | 4 |
| 6 | Edema | 2 | 2.9 | 6 | 1 | 1.1 | 7 | 1 | 2.3 | 6 |
| 7 | Dizziness | 0 | 0.0 | 7 | 2 | 2.2 | 6 | 1 | 2.3 | 6 |
