# Supplementary figures and images for: Patients' Preferences for Parkinson's Disease Pharmacotherapy: An Online Discrete Choice Experiment
Source: Parkinsons Dis. 2025 Jul 29;2025:9526138. doi: 10.1155/padi/9526138 (PMC12324919; doi:10.1155/padi/9526138)

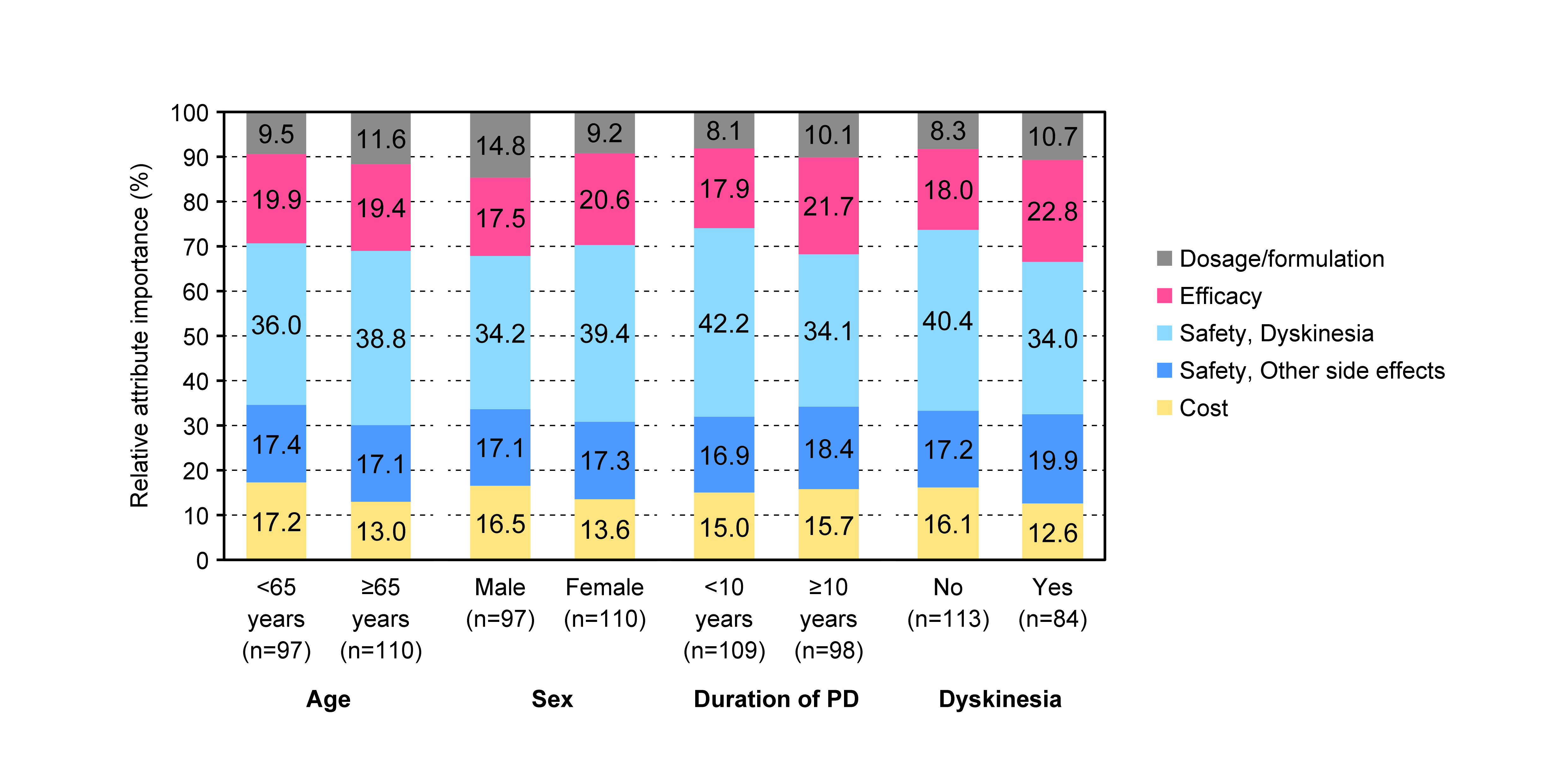

Supplement: Supporting Information 8 — Supporting Figure 1: Relative attribute importance according to various demographic and clinical characteristics. [file 9526138.f8.zip › Supplementary Figure 1.tif]

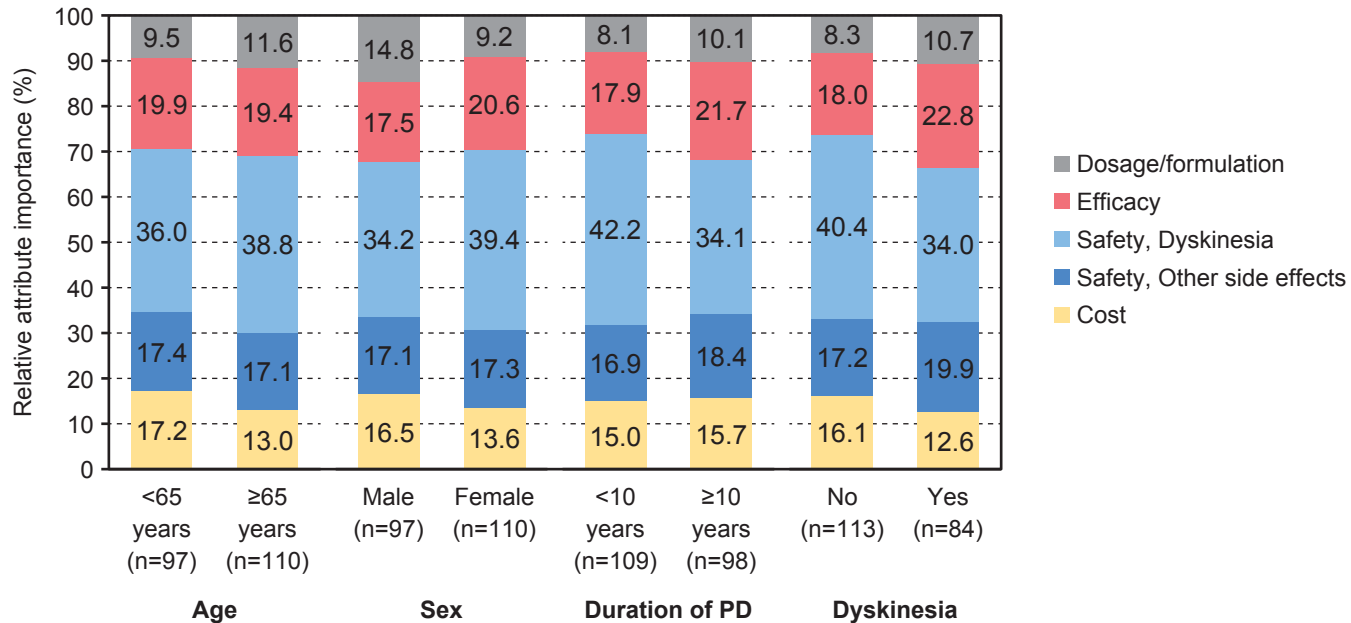

Supplement: Supporting Information 8 — Supporting Figure 1: Relative attribute importance according to various demographic and clinical characteristics. [file 9526138.f8.zip › Supplementary Figure.pdf]
